# Longitudinal Zeolite-Iron Oxide Nanocomposite Deposited Capacitance Biosensor for Interleukin-3 in Sepsis Detection

**DOI:** 10.1186/s11671-021-03527-w

**Published:** 2021-04-26

**Authors:** Chao Chen, Subash C. B. Gopinath, Periasamy Anbu

**Affiliations:** 1 Department of Intensive Care Units, Henan Provincial People’s Hospital, Zhengzhou University People’s Hospital, Henan University People’s Hospital, Zhengzhou, 450000 Henan China; 2 Institute of Nano Electronic Engineering, Universiti Malaysia Perlis (UniMAP), 01000 Kangar, Perlis, Malaysia; 3 Faculty of Chemical Engineering Technology, Universiti Malaysia Perlis (UniMAP), 02600, Arau, Perlis, Malaysia; 4 Department of Biological Engineering, College of Engineering, Inha University, Incheon, 402-751 Republic of Korea

**Keywords:** Sepsis, Interleukin-3, Nanobiosensor, Immunoassay, Biomarker

## Abstract

Sepsis is an extreme condition involving a physical response to severe microbial infection and causes fatal and life-threatening issues. Sepsis generates during the chemicals release with the immune system into the bloodstream for fighting against an infection, which causes the inflammation and leads to the medical emergency. A complexed longitudinal zeolite and iron oxide nanocomposite was extracted from coal mine fly ash and utilized to improve the surface characteristics of the capacitance biosensor to identify sepsis attacks. Anti-interleukin-3 (anti-IL-3) antibody was attached to the zeolite- and iron oxide-complexed capacitance electrode surface through an amine linker to interact with the sepsis biomarker IL-3. The morphological and chemical components of the nanocomplex were investigated by FESEM, FETEM, and EDX analyses. At approximately 30 nm, the longitudinal zeolite and iron oxide nanocomposite aided in attaining the limit of IL-3 detection of 3 pg/mL on the linear curve, with a regression coefficient (*R*^2^) of 0.9673 [*y* = 1.638*x* − 1.1847]. A lower detection limit was achieved in the dose-dependent range (3–100 pg/mL) due to the higher amount of antibody immobilization on the sensing surface due to the nanomaterials and the improved surface current. Furthermore, control experiments with relevant biomolecules did not show capacitance changes, and spiked IL-3 in human serum increased capacitance, indicating the specific and selective detection of IL-3. This study identifies and quantifies IL-3 via potentially useful methods and helps in diagnosing sepsis attack.

## Introduction

Sepsis is a fatal condition that occurs when the body responds severely to an infection [1]. Due to sepsis attack, the body produces a higher level of signaling biomolecules called ‘cytokines’, which attract immune cells. Increasing numbers of these cells secrete more cytokines, and the cytokine storm recruits more immune cells. Instead of controlling the initial infection, immune factors attack body organs and tissues. Furthermore, this infection triggers a chain reaction in the whole body and causes organ failure and tissue damage [2]. In particular, sepsis starts in the lungs, skin, urinary tract and gastrointestinal tract and spreads widely.

to other organs, which leads to organ injury. Therefore, it is necessary to stop the process earlier to prevent attacks on other organs. Sepsis identification in its earlier stages with a suitable biomarker helps provide prompt treatment to patients and save lives. Researchers found that the interleukin-3 (IL-3) inflammatory factor is an independent predictor of sepsis attack and death produced by innate response activator (IRA) B cells following Toll-like receptor activation. Furthermore, it was found that a higher level of IL-3 was associated with a higher rate of mortality in sepsis patients and confirmed that IL-3 plays a major role in immune regulation and higher responses to corticosteroids during sepsis. This research aimed to quantify the level of IL-3 using a zeolite-iron oxide (zeolite-iron) nanocomposite-modified capacitance electrochemical sensor.

The detection of biomolecules with biosensors is highly dependent on the immobilization of the target or detection molecule on the transducer electrode surface [3]. A higher number of immobilized capture probes with a proper orientation leads to lower target detection limits [4]. In most cases, capture probe immobilization has been conducted through physical adsorption, electrostatic interaction, covalent linking, and biomolecule entrapment with polymers [5, 6]. It may be difficult to achieve reproducibility and biocompatibility of immobilized biomolecules by the above immobilization methods. In particular, the attachment of smaller capture molecules such as RNA, DNA, aptamers, and peptides to sensing surfaces is complicated [7, 8]. Various researchers have used different techniques for efficient immobilization. Recently, nanomaterials have been highly attractive for use in the process of biomolecular immobilization on sensing surfaces [9–11]. Gold nanoparticles have been efficiently immobilized on various sensing surfaces, such as polystyrene ELISA substrates and aluminum electrodes, and have captured potential molecules, including antibodies, proteins, DNA and aptamers. In addition to silica, aluminum and graphene are commonly used materials for the immobilization of biomolecules. These nanomaterials improve the number of capture molecules and yield the biocompatibility and stability of the biomolecules on sensing surfaces, which helps improve the detection strategy. Recently, inorganic materials such as zeolite, clay, and sol–gel have attracted the attention of researchers for solving these problems. Moreover, the synthesis of nanomaterials by greener approaches has been highly encouraged due to great associated advantages, such as not involving hazardous materials and being less expensive [12–15]. Furthermore, tailored approaches can be used to achieve desired sizes and shapes of nanomaterials. The current study was in line with this approach and produced a zeolite nanomaterial combined with isolated iron to create a nanocomposite.

Zeolite is a crystalline microporous aluminosilicate composed of TO_4_ tetrahedra and O atoms [16]. It is a promising material for biomolecular immobilization due to its larger surface area, capability for ion exchange, controllable hydrophobicity/hydrophilicity, and high mechanical and thermal conductivity. Therefore, various studies in the biosensor field have been established using zeolite material for the biomolecular immobilization process and reached lower detection limits to develop biosensors such as glucose sensors and urea sensors [17–19]. Similarly, iron nanomaterials improve the effect of cation exchange properties and give quick responses to current changes upon interactions with biomolecules. The zeolite-iron oxide nanocomposite with a unique composition used in this study enhances the high performance of the sensor, further providing improved sensitivity due to the improvement in conductivity. This study focused on zeolite-iron nanomaterials extracted from coal mine fly ash and utilized them as substrates for capacitance electrodes to immobilize the capture probe, which was anti-IL-3 antibody. Compared to previous nanomaterial-mediated detections of different interleukins [20–22], the current sensing system provides improvements in several ways. For instance, its higher suitability for electrochemical sensors requires fewer experimental steps, and it displays suitability for surface chemical functionalization and a high nonfouling capability.

A capacitive biosensor is an electrochemical sensor created by registering the attraction between the probe and target interacting on the electrode surface. A capacitive biosensor helps measure changes in the dielectric layer at the electrode interface when a target biomolecule is binding with the immobilized probe on the sensing surface [23]. The capacitance between the electrode and the electrolyte is described by *C* = (2*nε*_0_*εA*)/*d*, where *ε*: dielectric constant, *A*: surface area of the plate, *ε*_0_: permittivity of the free space, *n*: number of repetitive fingers, and *d*: insulating layer thickness [24, 25]. A nonfaradic capacitive biosensor helps avoid protein denaturing caused by metallization and improves the interaction of target and probe binding [26, 27]. Various studies have utilized capacitive biosensors to identify various target molecules, including nucleotides, heavy metals, proteins and organic molecules [23, 28–31]. In this research, a nonfaradic capacitance biosensor experiment was conducted on a zeolite-iron-modified electrode surface. Zeolite-iron was attached to the capacitance electrode through (3-aminopropyl)-trimethoxysilane as an amine linker, and then anti-IL-3 was attached to the surface through the attraction between the amine surface and the carboxylic (COOH) group of the antibody. An anti-IL-3 antibody-modified electrode was used to quantify IL-3 and diagnose sepsis attack.

## Materials and Methods

### Apparatus and Reagents

Fly ash was received from a thermal power plant in India. Sodium hydroxide and sulfuric acid were purchased from Sigma Aldrich (Missouri, USA). (3-Aminopropyl)-trimethoxysilane (APTMS) was received from Merck (NJ, USA). Whatman filter paper was obtained from Thermo Fisher Scientific (Massachusetts, USA). IL-3 and anti-IL-3 antibodies were received from Santa Cruz Biotechnology (Texas, USA). Field-emission scanning electron microscopy (FESEM; Hitachi, S-4300 SE, Japan) and field-emission transmission electron microscopy (FETEM; JEM-2100F, JEOL, Japan) were used to analyze the zeolite-iron nanomaterial by methods outlined previously [32].

### Synthesis of Zeolite-Iron Nanomaterial

Zeolite-iron nanomaterials were synthesized using iron, silica, and alumina extracted from fly ash. The following three major steps were involved in this procedure: (1) separation of iron particles; (2) extraction of sodium aluminosilicate; and (3) preparation of zeolite-iron nanoparticles by the sol–gel synthesis method.

### Separation of Iron from Fly Ash

A 25 g aliquot of fly ash was mixed with 500 µL of distilled water and stirred for 30 min using a magnetic stirrer. The iron particles stuck to the magnetic stirrer were separated, and then 1 g of the separated particles was mixed with 25% sulfuric acid and stirred for 1 h at 50 °C. Then, the iron-containing solution and the iron particles were separated by Whatman filter paper and utilized as the base for iron oxide to synthesize the zeolite-iron nanomaterial.

### Extraction of Sodium Aluminosilicate from Fly Ash

The alkaline extraction method was used to extract sodium aluminosilicate from fly ash by the alkaline extraction method [33]. First, 25 g of iron-separated fly ash was mixed with 500 µL of 2 M sodium hydroxide and heated at 100 °C with stirring for 6 h. After cooling the solution, sodium aluminosilicate (the solution in the mixture) was separated by Whatman filter paper. The filtered solution was used as the base to synthesize a zeolite.

### Zeolite-Iron Nanomaterial Synthesized by the Sol–Gel Method

The extracted iron and sodium aluminosilicate were used as the base to synthesize zeolite-iron nanomaterials by the sol–gel method. In the first step, a beaker containing 200 mL of sodium aluminosilicate at pH 12 was placed on the hotplate under stirring. Then, the solution was titrated with iron solution at pH 1 by adding dropwise amounts of iron solution until it reached pH 7. At pH 7, a white gel was formed, and this gel was continuously stirred overnight to obtain uniformly distributed zeolite-iron nanoparticles. The next day, the gel was separated by centrifugation (10,000×*g* for 10 min) and then washed with 25% ethanol and distilled water. The final product was dried at 100 °C for 1 h to obtain a powder consisting of zeolite-iron nanomaterials. The surface of the zeolite-iron nanomaterial was characterized by FETEM and FESEM. Energy-dispersive X-ray (EDX) analysis was also conducted to identify the elements in the zeolite-iron.

### Amine Modification of Zeolite-Iron and Functionalization of the Capacitance Electrode Surface

Amine was coated onto the surface of the zeolite-iron by the silane coupling agent APTMS. For this, 1 g of zeolite-iron was mixed with 1% KOH for 10 min, and then the excess KOH was removed by distilled water. Afterward, the KOH-treated zeolite-iron was mixed with 1% APTMS, and the mixture was placed on a heated stirrer overnight. The next day, the nanomaterial was washed with ethanol and separated by centrifugation (10,000×*g* for 10 min). This APTMS-zeolite-iron was attached to the capacitance electrode surface to identify IL-3. For this immobilization, APTMS-zeolite-iron was dropped on the hydroxylated electrode and kept at RT for 3 h. The bonding between the zeolite-iron oxide nanocomposite, APTMS and sensing surface was due to silane coupling with the generated oxide groups. In general, silane coupling occurs with multiple arms available to the oxide groups, yielding linkages among the zeolite-iron oxide nanocomposite, APTMS and sensing surface. Due to these multiple linkages, a spatial arrangement on the sensing surface is formed. After washing the surface with ethanol followed by water, an anti-IL-3 antibody immobilization process was performed to interact with IL-3.

### Determination of IL-3 on the Anti-IL-3-modified Capacitance Electrode Surface

The surface described above was formed by amine tethering, which allows reaction with available COOH groups when the antibody attaches. IL-3 was identified on an anti-IL-3 immobilized capacitance electrode surface. For this, IL-3 at 100 pg/mL was diluted in PBS buffer and dropped onto an antibody-modified electrode surface. Following antibody immobilization, the remaining unbound surface was blocked by PEG-COOH (1 mg/ml). A similar reaction to antibody-APTMS occurs when PEG-COOH is attached. The capacitance value was recorded before and after interacting with IL-3. Differences in value were considered for the binding of IL-3 with its antibody. Furthermore, to calculate the limit of detection, IL-3 was titrated from 3 to 50 pg/mL and dropped independently onto the antibody-modified surfaces. The other experimental procedure was performed as described previously. The difference in capacitance value for each IL-3 concentration was calculated and plotted to calculate the detection of IL-3 by the R^2^ value. When IL-3 is attached to the antibody immobilized surface, there will be a genuine interaction.

### Biofouling and Selective Determination of IL-3 on the Zeolite-Iron Modified Capacitance Electrode Surface

A biofouling experiment was conducted under three different biomolecule conditions, including the presence of a nonimmune antibody or a control protein and the absence of IL-3 antibody. In the first case, instead of IL-3 antibody, a nonimmune antibody was used; in the second experiment, instead of IL-3, a control protein was used; and the last experiment was conducted without IL-3 antibody. The capacitance values were compared for the specific interaction of IL-3 antibody with IL-3. A selective experiment was conducted by spiking IL-3 in a 1:100 dilution of human serum and adding it dropwise onto an anti-IL-3-modified electrode surface. The changes in capacitance were recorded for each IL-3 concentration to identify the selective identification of IL-3. Reproducibility was confirmed by repeating the experiments three times (in triplicate) with similar devices made from the same batch fabrication. The lifetime storage and stability of the probe-immobilized surface were also determined.

## Results and Discussion

**Fig. 1:**
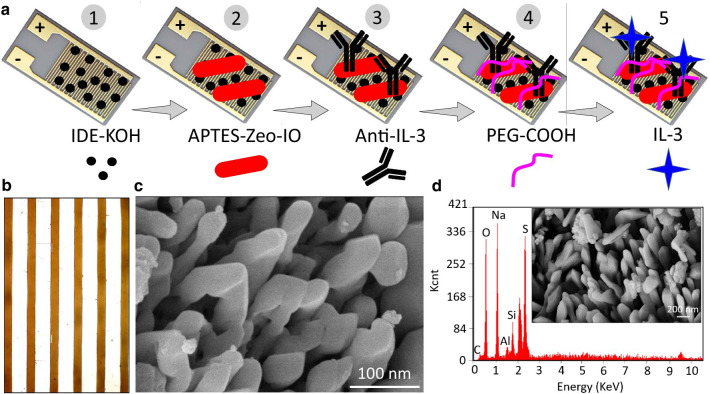
**a** Schematic illustration of IL-3 identification on an anti-IL-3-modified capacitance sensing surface. APTMS-modified zeolite-iron was used to attach the capture antibody and then interacted with IL-3. PEG-COOH was used as the blocking agent. **b** Morphological analysis of the capacitance sensing surface by high-power microscopy. **c** Morphological analysis of zeolite-iron by FESEM. Nanomaterials were formed with a uniform distribution and in the longitudinal dimension. **d** EDX analysis. The presence of the main elements Si, Al, Fe, and O was found. The figure inset was obtained by FESEM with low magnification

Sepsis is the condition with lot of chemicals released in the immune system, and triggers a widespread inflammation with the ultimate damage of the organs. Figure 1a shows a schematic illustration of IL-3 identification on an anti-IL-3-modified capacitance electrode surface for determining the condition associated with sepsis. APTMS-modified zeolite-iron was used to attach the capture antibody to the sensing electrode surface. APTMS on the surface of the zeolite-iron was immobilized on the electrode surface through the interaction between amine on the nanomaterial and OH groups on the electrode surface. Figure 1b confirms the intactness of the surface electrode on the capacitance sensor, as captured under a high-power microscope. The antibody was linked to the surface through COOH and the amine of the zeolite-iron. Zeolite-iron helps attach a higher number of APTMS on the sensing electrode, which helps capture more antibodies on the capacitance electrode surface. Various studies have indicated that capture probe immobilization on the sensing surface plays a crucial role in lowering the detection limit of the target molecule. In this research, the zeolite-iron nanomaterial was used to attach anti-IL-3 antibody to the sensing electrode surface. Higher immobilization of anti-IL-3 antibody with proper orientation helps reach the lower detection limits of IL-3.

### Morphological Analysis of Zeolite-Iron Nanomaterial by FESEM and FETEM

**Fig. 2:**
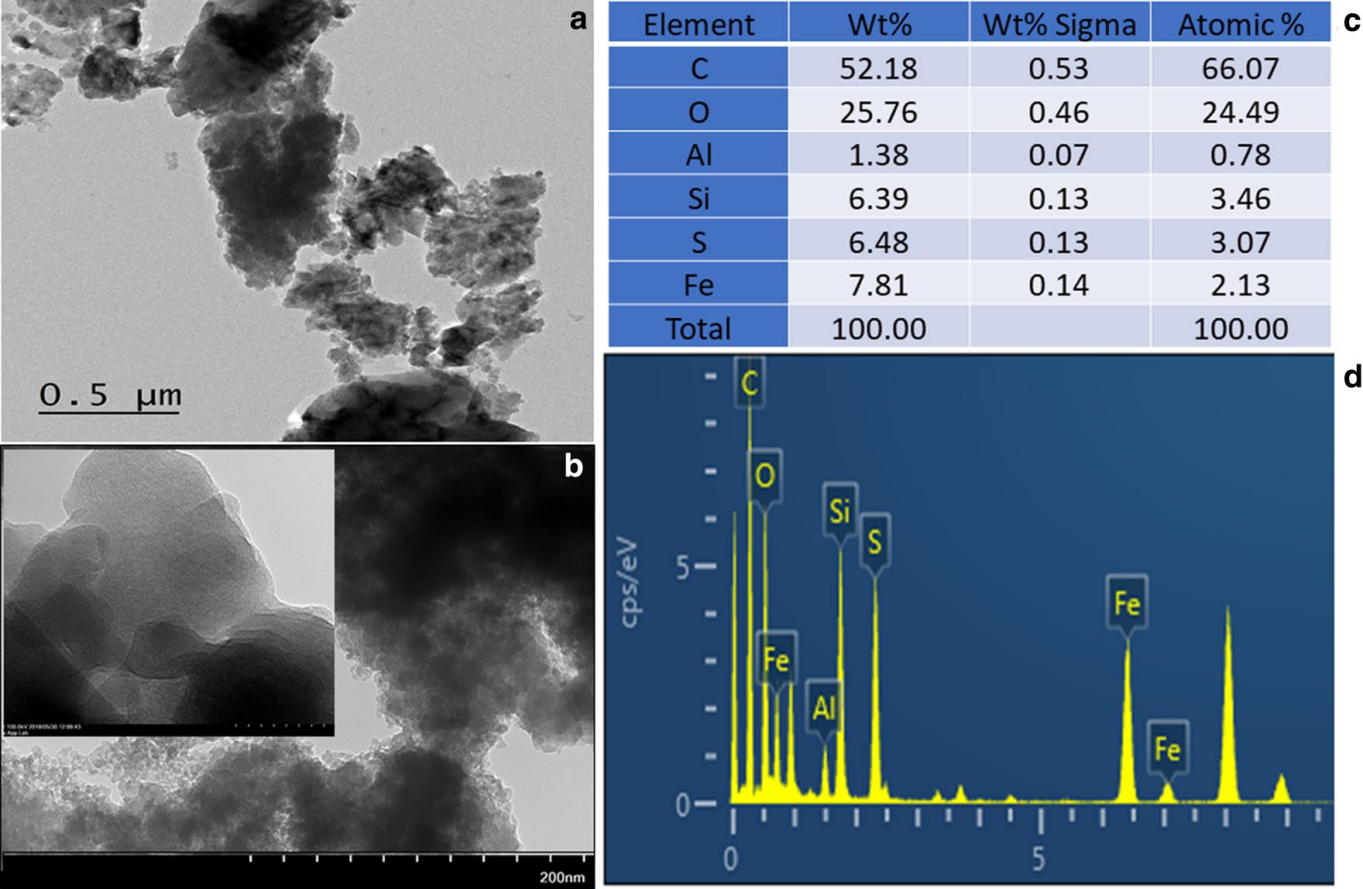
FETEM images of zeolite-iron at the **a** 50 nm scale and **b** 200 nm scale. The nanomaterial was formed with a uniform distribution. **c** EDX analysis confirming the presence of the main elements Si, Al, Fe, C, and O

Figure 1c, d (inset) displays the morphological image of the zeolite-iron nanomaterial obtained from FESEM at different magnifications and the EDX elemental analysis. The obtained zeolite-iron nanomaterial was smooth and uniformly distributed, with a densely stacked longitudinal nanostructure. The size of this nanostructure was ~ 30 nm, and the obtained image shows that the nanocomposite was arranged with uniform shapes and distributed well at the proper distance. The FETEM image also proved a shape similar to that obtained by FESEM for the formed zeolite-iron nanocomposites (Fig. 2a, b). The EDX result confirmed the presence of Fe, Al, Si, and O in the synthesized zeolite-iron nanocomposite (Fig. 2c, d). The major elemental atomic percentages of Si, Al, Fe and O were found to be 3.46, 0.78, 2.13 and 24.39%, respectively. This FESEM and EDX result confirms the formation of zeolite-iron nanomaterials.

### Sensing Electrode Preparation for IL-3 Determination

**Fig. 3:**
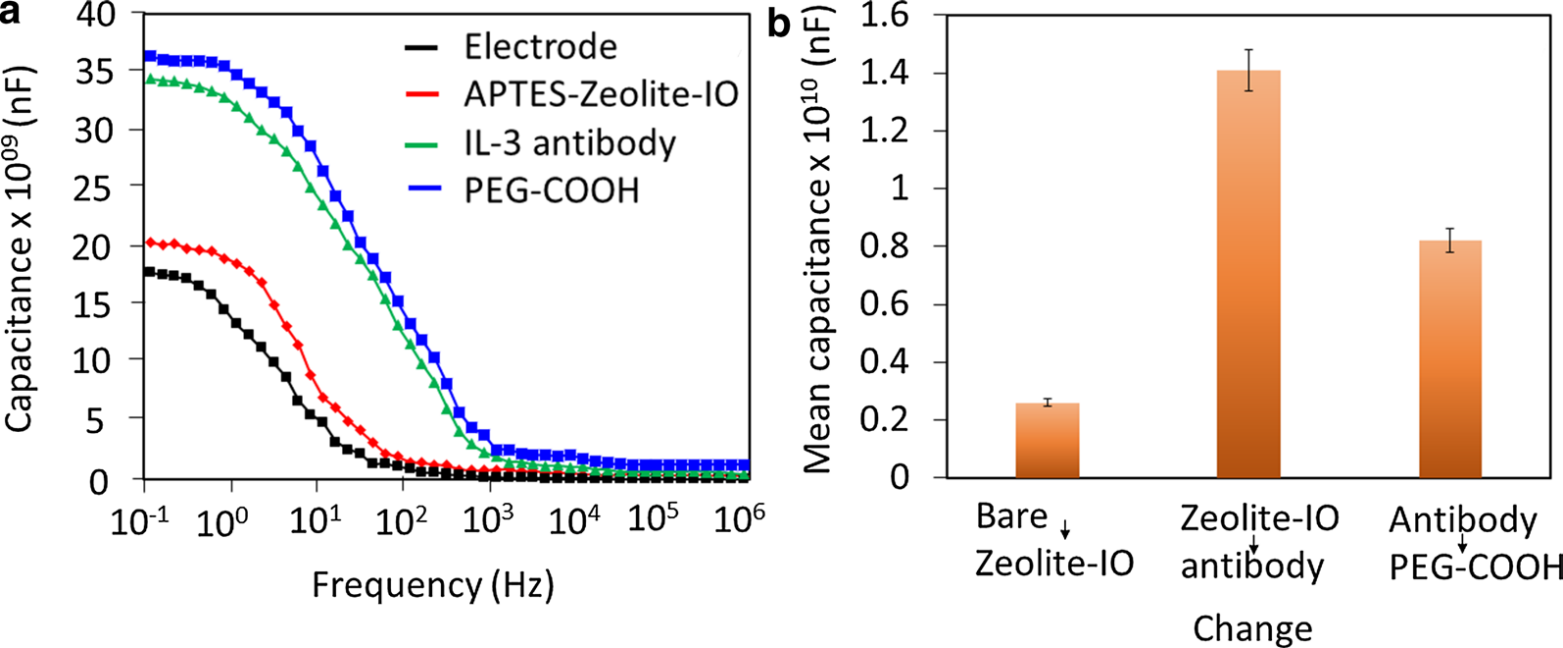
**a** Process of antibody attachment to the zeolite-iron-modified surface. capacitance values increased after each molecular immobilization. **b** Difference in capacitance. Anti-IL-3 antibody immobilization showed a higher change in the capacitance value. The values were averaged using three readings as triplicates. [Zeolite-IO—Zeolite-iron oxide]

Anti-IL-3 antibody immobilization on the capacitance biosensor was confirmed by altering the level of capacitance after each biomolecular immobilization. Figure 3a shows the capacitance changes during the process of antibody attachment to the zeolite-iron-modified surface. As shown in Fig. 3a, the KOH-tethered capacitance electrode surface shows a capacitance value of 1.74 × 10^09^ nF, and after dropwise addition of the APTMS-zeolite-iron, it was increased to 2.02 × 10^09^ nF. This increase in capacitance confirmed the attachment of the nanomaterials to the sensing electrode. Furthermore, upon adding anti-IL-3 antibody, the capacitance value increased drastically to 3.42 × 10^09^ nF. This higher increment was noted due to the higher amount of antibody immobilization on the APTMS-modified zeolite-iron. Additionally, the nanomaterials showed a proper arrangement with a higher number of APTMS on the sensing electrode surface, ultimately attracting more antibodies. Finally, PEG-COOH was added for blocking purposes, and the capacitance was found to increase to 3.64 × 10^09^ nF. The sensor works based on changes in surface charge and, ultimately, changes in capacitance. The surface charge changes vary with molecular attachments/interactions. Each molecule carries different charges and influences the capacitance of the sensor. Therefore, the difference in the capacitance was considered for the measurements. Higher changes in capacitance were noted due to the higher occupancy of IL-3 antibodies on the sensing electrode surface (Fig. 3b). PEG-COOH helps reduce nonspecific binding of IL-3 on the sensing electrode surface and eliminates false-positive results. Various studies have proven that PEG-based polymers on sensing surfaces improve biocompatibility, reduce the signal-to-noise ratio and provide a proper orientation of immobilized biomolecules on sensing surfaces, which leads to a lower detection limit of the sensor [34–37]. Since the APTMS surface attracts other biomolecules electrostatically, PEG-COOH was used to cover the excess APTMS surface on the zeolite-iron nanomaterial, which helps reduce biofouling. This anti-IL-3 modified surface was utilized to identify IL-3.

### Determination and Quantification of IL-3 on the Anti-IL-3 Antibody Surface

**Fig. 4:**
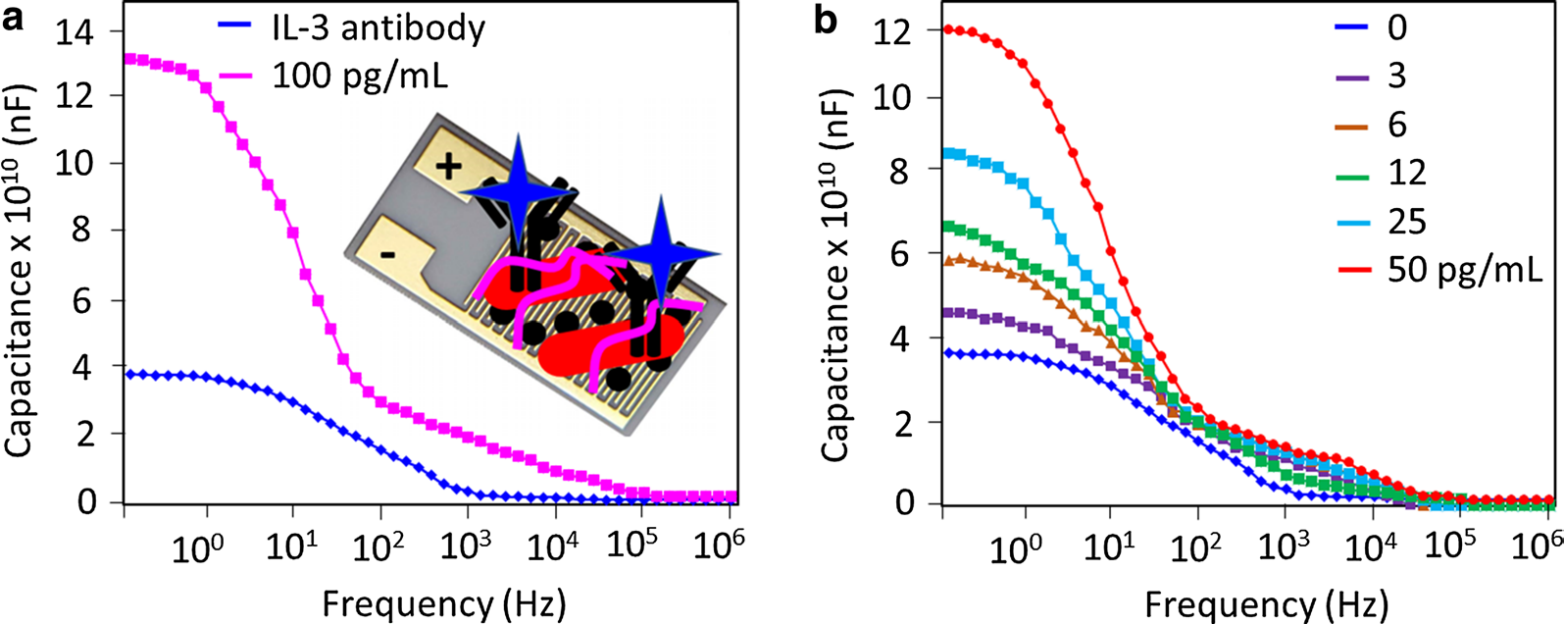
Determination of IL-3 on the anti-IL-3-modified surface. **a** Identification of 100 pg/mL of IL-3. Clear changes in capacitance were noted after dropwise addition of IL-3. The figure inset displays the schematic. **b** Titration of different IL-3 concentrations onto anti-IL-3 antibody. With all concentrations of IL-3, capacitance changes were noted

**Fig. 5:**
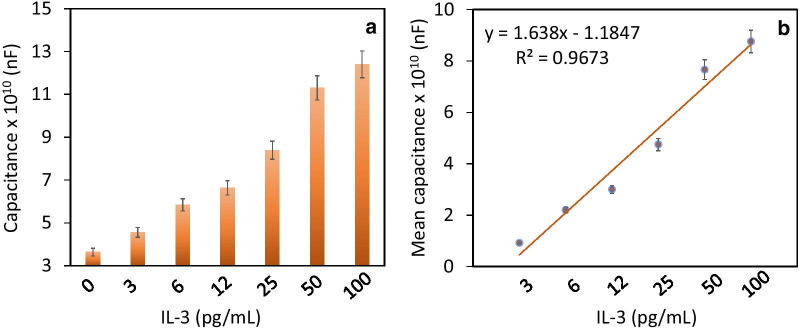
**a** Capacitance value for each IL-3 concentration. Increasing the IL-3 concentration produced a gradual increase in the capacitance value. **b** Difference in the capacitance value for each IL-3 concentration. The values were plotted in an Excel sheet, and the detection limit of IL-3 was calculated as 3 pg/mL. The values were averaged using three readings as triplicates

IL-3 was quantified on an anti-IL-3-modified capacitance electrode sensing surface. Initially, a higher concentration of IL-3 (100 pg/mL) was tested on the antibody-modified surface, and the capacitance value increased from 3.74 × 10^10^ nF to 13 × 10^10^ nF. This increase confirms the interaction of IL-3 with anti-IL-3 antibody (Fig. 4a). A similar experiment was conducted with IL-3 concentrations of 3 to 50 pg/mL. As shown in Fig. 4b, the capacitance values increased to 4.56 × 10^10^ nF, 5.84 × 10^10^ nF, 6.64 × 10^10^ nF, 8.39 × 10^10^ nF, and 12 × 10^10^ nF. It was noted that with increasing concentrations of Il-3, the capacitance values gradually increased (Fig. 5a). The difference in the capacitance values was calculated and plotted in an Excel sheet, and the detection of IL-3 was calculated as 3 pg/mL with an R2 value of 0.9673 (Fig. 5b). A linear dose-dependent response with different concentrations of IL-3 (3–100 pg/mL) was found when interacting with anti-IL-3. However, when titrated at further concentrations, the samples were saturated.

### Biofouling/Nonfouling on the APTES-Zeolite-Iron-Modified Capacitance Electrode

**Fig. 6:**
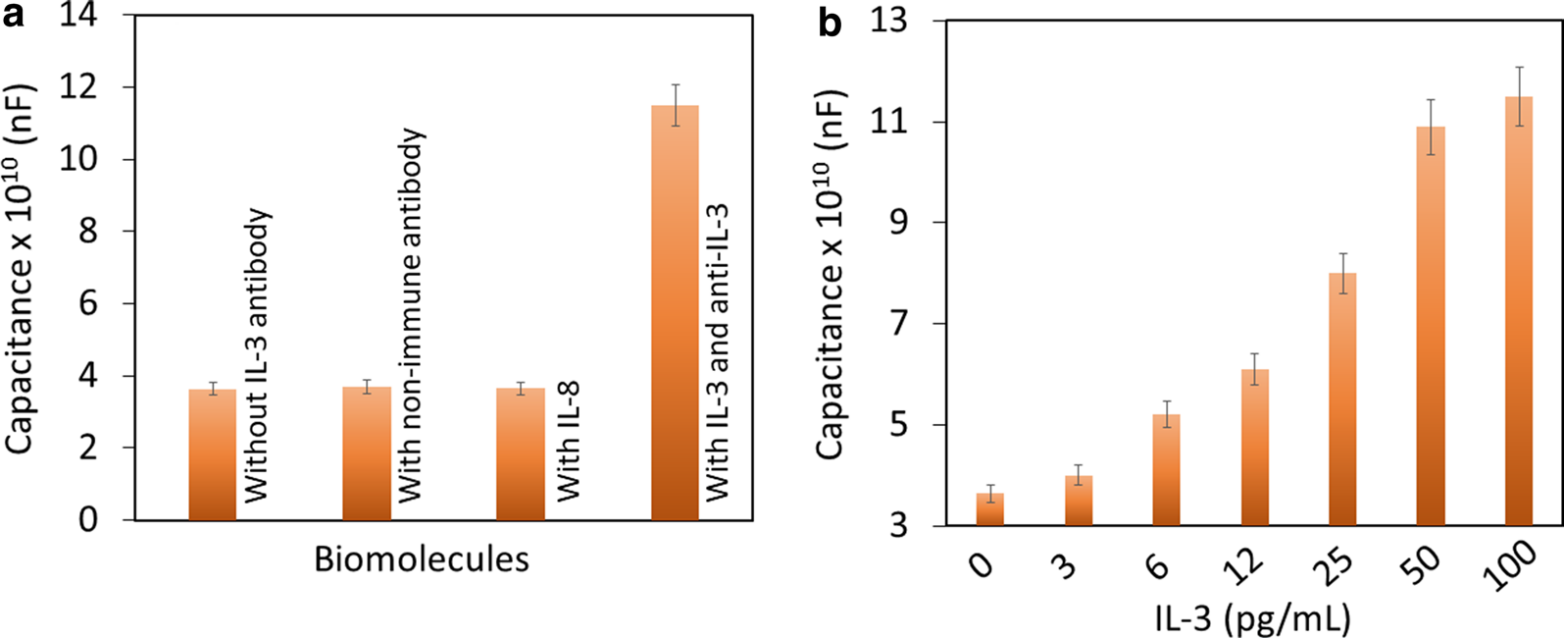
**a** Specific detection of IL-3. Control molecules did not show an increase in capacitance value, indicating the specific detection of IL-3. **b** Spiking of IL-3 in human serum. Spiking in human serum increased the capacitance with increasing IL-3 concentrations. This result confirms the selective detection of IL-3. The values were averaged using three readings as triplicates

Biofouling is the biggest issue in any kind of biosensor, which leads to false-positive identification of the target on the sensing surface. Blocking agents such as BSA, ethanolamine and PEG-based polymers are common molecules that reduce biofouling on sensing surfaces. Herein, PEG-COOH was used as the blocking agent, and the biofouling effect was confirmed in three different control experiments, namely, without IL-3 antibody, with a nonimmune antibody and with a control protein (IL-8). As shown in Fig. 6a, all three control experiments failed to enhance the capacitance value, indicating the specific identification of IL-3 without any biofouling.

### Spiking of IL-3 in Human Serum and Stability

Different concentrations of IL-3 were spiked in human serum and subjected to the same experimental procedure to identify IL-3 in real-life situations. As displayed in Fig. 6b, spiked IL-3 in human serum clearly produced increased capacitance values with enhanced concentrations of IL-3. This result confirms selective IL-3 identification by the anti-IL-3-modified capacitance electrode.

Considering the reproducibility, the sensing surface behaves well, with minimal error values. The operational lifetime of the fabricated sensing surface-attached nanomaterial can be prolonged for three months with proper storage in a desiccator. However, after attaching the probe, the surface was stable for 2 weeks and tended to lose 19% of the stability, and the loss of stability became steep beginning at the 3rd week. To prove the high performance of the current sensor, a comparison study has been performed with the currently available sensors, and the results indicate that it is comparable and behaves better in several instances (Table 1).

**Table 1:** Comparison among currently available sensing systems for interleukins

Biosensor	Target	Probe	Detection limit	References
Optic biosensor	IL-6	–	5 pM	[38]
Plasmonic sensor	IL-6	Antibody	0.1 pg/mL	[20]
ELISA	IL-10	Antibody	1–15 pg/mL	[39]
Impedance biosensor	IL-8	Synthetic binding protein	90 fg/mL	[40]
Field effect transistor	IL-6	–	1.53 pg/mL	[21]
Chemiluminescent biosensor	IL-5	Antibody	0.1 pg/mL	[41]
Fluorescence assay	IL-6	Antibody	11.859 pg/mL	[42]
Electrochemical sensor	IL-8	Stem loop probe	200 pM	[22]
Wearable nonfaradaic sensor	IL-6	Antibody	0.2 pg/mL	[43]
Impedance spectroscopy	IL-3	Antibody	3 pg/mL	This work

## Conclusion

Sepsis is life-threatening, involves an overwhelming immune reaction, is extremely dangerous and affects the whole body. This study demonstrated the identification of a sepsis biomarker (IL-3) on a capacitance electrode. Zeolite-iron nanomaterial was extracted from a coal fly-modified capacitance electrode to increase the current flow upon binding of biomolecules to the sensing electrode. Amine modification was carried out to attach anti-IL-3 antibody to the zeolite-iron. IL-3 detection was conducted on an antibody-modified electrode and reached the detection limit of IL-3 to 3 pg/mL. Further control experiments failed to show an increase in capacitance value, confirming the specific detection of IL-3, and selective experiments with spiked IL-3 in human serum displayed a clear increase in capacitance. This experimental method quantifies IL-3 levels and helps diagnose sepsis attacks.

## Data Availability

All of the data are fully available without restriction.

## Abbreviations

IL-3: Interleukin-3
pg: Picogram
mL: Milliliter
µL: Microliter
M: Molar
FESEM: Field-emission transmission electron microscope
FETEM: Field-emission scanning electron microscope
EDX: Energy-dispersive X-ray
IRA: Innate response activator
COOH: Carboxylic
DNA: Deoxyribose nucleic acid
RNA: Ribose nucleic acid
ELISA: Enzyme-linked immunosorbent assay
APTMS: (3-Aminopropyl)-trimethoxysilane
KOH: Potassium hydroxide
PEG: Polyethylene glycol
